# 7-Bromo-3,3-dibutyl-8-meth­oxy-2,3-di­hydro-1,5-benzothia­zepin-4(5*H*)-one

**DOI:** 10.1107/S1600536813013238

**Published:** 2013-06-19

**Authors:** C. V. Deepu, M. Manjula, K. J. Pampa, D. G. Bhadregowda, N. K. Lokanath

**Affiliations:** a Department of Chemistry, Yuvaraja’s College, University of Mysore, Mysore 570 006, India; b Department of Studies in Physics, Manasagangotri, University of Mysore, Mysore 570 006, India; c Department of Studies in Microbiology, Manasagangotri, University of Mysore, Mysore 570 006, India

## Abstract

In the title compound C_18_H_26_BrNO_2_S, the thia­zepine ring adopts a boat conformation. The dihedral angle between the mean planes through the benzene ring and the four C atoms making up the basal plane of the boat is 35.8 (2)°. In the crystal, inversion dimers linked by pairs of N—H⋯O hydrogen bonds generate *R*_2_^2^(8) loops.

## Related literature

For reference bond lengths, see: Allen *et al.* (1987 ▶). For background to the uses of this class of compounds, see: Fedi *et al.* (2008 ▶); Ganesh *et al.* (2011 ▶); Riedel *et al.* (2007 ▶).
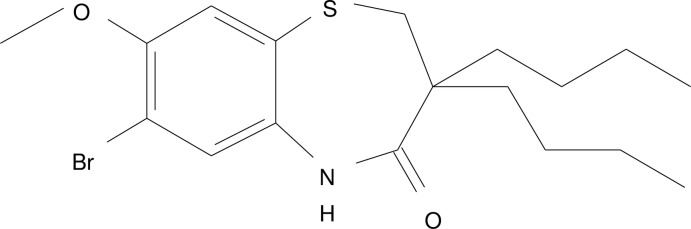


## Experimental

### 

#### Crystal data

C_18_H_26_BrNO_2_S
*M* *_r_* = 400.30
Monoclinic, 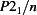

*a* = 7.7844 (18) Å
*b* = 11.251 (2) Å
*c* = 22.039 (6) Å
β = 98.199 (8)°
*V* = 1910.5 (8) Å^3^
*Z* = 4
Mo *K*α radiation
μ = 2.27 mm^−1^
*T* = 100 K
0.32 × 0.20 × 0.20 mm

#### Data collection

Oxford Diffraction Xcalibur Eos diffractometer
16056 measured reflections
4662 independent reflections
2750 reflections with *I* > 2σ(*I*)
*R*_int_ = 0.054

#### Refinement

*R*[*F* ^2^ > 2σ(*F* ^2^)] = 0.048
*wR*(*F* ^2^) = 0.141
*S* = 1.03
4662 reflections
212 parameters
H-atom parameters constrained
Δρ_max_ = 0.63 e Å^−3^
Δρ_min_ = −0.49 e Å^−3^

### 

Data collection: *CrysAlis PRO* (Oxford Diffraction, 2009 ▶); cell refinement: *CrysAlis PRO*; data reduction: *CrysAlis PRO*; program(s) used to solve structure: *SHELXS97* (Sheldrick, 2008 ▶); program(s) used to refine structure: *SHELXL97* (Sheldrick, 2008 ▶); molecular graphics: *Mercury* (Macrae *et al.*, 2006 ▶); software used to prepare material for publication: *Mercury*.

## Supplementary Material

Crystal structure: contains datablock(s) global, I. DOI: 10.1107/S1600536813013238/hb7081sup1.cif

Structure factors: contains datablock(s) mm. DOI: 10.1107/S1600536813013238/hb7081Isup2.hkl

Supplementary material file. DOI: 10.1107/S1600536813013238/hb7081Isup3.cml

Additional supplementary materials:  crystallographic information; 3D view; checkCIF report

## Figures and Tables

**Table 1:** Hydrogen-bond geometry (Å, °)

*D*—H⋯*A*	*D*—H	H⋯*A*	*D*⋯*A*	*D*—H⋯*A*
N14—H14⋯O15^i^	0.86	2.13	2.985 (3)	175

Symmetry code: (i) 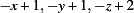.

**Fig. 1.**
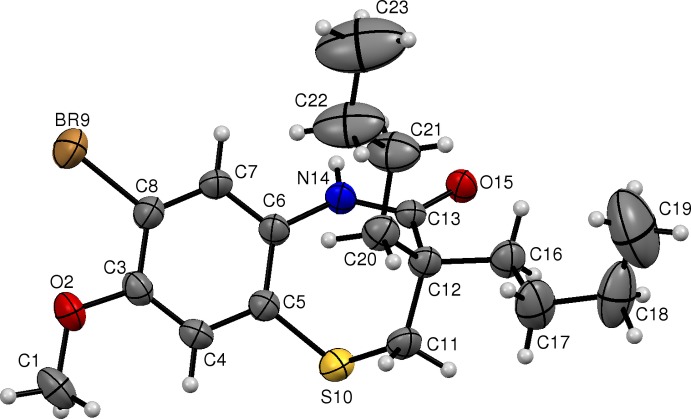
View of the title molecule with 50% probability ellipsoids.

**Fig. 2.**
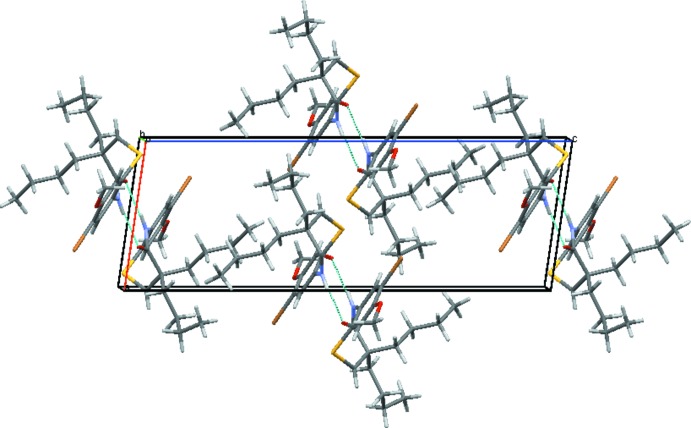
Packing diagram of molecule, viewed along the crystallographic b axis.

## supplementary crystallographic information

### Comment

The title compound C_18_H_25_Br_N_O_2_S, was synthesized from 3,3-dibutyl-2,3- dihydro-8-methoxybenzo[*b*][1,4]thiazepin-4(5*H*)-one. The molecule is a bicyclic structure with one aromatic ring fused to a seven membered ring, on which two heteroatoms are present. The derivatives of this molecule able to provide high affinity ligands for more than one type of the receptor (Fedi *et al.*, 2008). The compound is mainly used to treat schizophrenia and also find applications as neuroleptics, antidepressants, antihistaminic (Ganesh *et al.*, 2011; Riedel *et al.*, 2007). The N14—C13 bond is shorter than an usual N—C single bond [1.356 Å compared to 1.416 Å (Allen *et al.* 1987)]. The atoms C5, C6, C11 and C12 present in the central thiazepine ring forms a basal plane and the S10 atom as the bow, representing the boat conformation of thiazepine ring.

### Experimental

3,3-dibutyl-2,3-dihydro-8-methoxybenzo[*b*][1,4]thiazepin-4(5*H*)-one in dichloromethane, acetonitrile, cooled to 5°C and added *N*-bromosuccinimide over a period of 15 min. Then reaction mixture was brought to room temperature, stirred for 2 h and again cooled to 5°C, then *N*-bromosuccinimide is added at 5°C, cooled to -5°C for 1 h, filtered, washed with cold acetonitrile and then dried in vacuum and product was recrystallized from acetonitrile solution to yield light brown blocks.

### Refinement

All hydrogen atoms were located geometrically with C—H = 0.93–0.97 Å and allowed to ride on their parent atoms with *U*_iso_(H) = 1.2*U*_eq_(aromatic C).

### Crystal data

**Table:**

C_18_H_26_BrNO_2_S	*F*(000) = 832
*M**_r_* = 400.30	*D*_x_ = 1.392 Mg m^−^^3^
Monoclinic, *P*2_1_/*n*	Mo *K*α radiation, λ = 0.71073 Å
Hall symbol: -P 2yn	Cell parameters from 4662 reflections
*a* = 7.7844 (18) Å	θ = 2.0–28.3°
*b* = 11.251 (2) Å	µ = 2.27 mm^−^^1^
*c* = 22.039 (6) Å	*T* = 100 K
β = 98.199 (8)°	Block, light brown
*V* = 1910.5 (8) Å^3^	0.32 × 0.20 × 0.20 mm
*Z* = 4	

### Data collection

**Table:**

Oxford Diffraction Xcalibur Eos diffractometer	2750 reflections with *I* > 2σ(*I*)
Radiation source: fine-focus sealed tube	*R*_int_ = 0.054
Graphite monochromator	θ_max_ = 28.3°, θ_min_ = 2.0°
Detector resolution: 16.0839 pixels mm^-1^	*h* = −10→8
ω scans	*k* = −14→9
16056 measured reflections	*l* = −29→29
4662 independent reflections	

### Refinement

**Table:**

Refinement on *F*^2^	Primary atom site location: structure-invariant direct methods
Least-squares matrix: full	Secondary atom site location: difference Fourier map
*R*[*F*^2^ > 2σ(*F*^2^)] = 0.048	Hydrogen site location: inferred from neighbouring sites
*wR*(*F*^2^) = 0.141	H-atom parameters constrained
*S* = 1.03	*w* = 1/[σ^2^(*F*_o_^2^) + (0.1275*P*)^2^ + 0.4493*P*] where *P* = (*F*_o_^2^ + 2*F*_c_^2^)/3
4662 reflections	(Δ/σ)_max_ = 0.001
212 parameters	Δρ_max_ = 0.63 e Å^−^^3^
0 restraints	Δρ_min_ = −0.49 e Å^−^^3^

### Special details

**Table:**

Geometry. Bond distances, angles etc. have been calculated using the rounded fractional coordinates. All su's are estimated from the variances of the (full) variance-covariance matrix. The cell esds are taken into account in the estimation of distances, angles and torsion angles
Refinement. Refinement on F^2^ for ALL reflections except those flagged by the user for potential systematic errors. Weighted R-factors wR and all goodnesses of fit S are based on F^2^, conventional R-factors R are based on F, with F set to zero for negative F^2^. The observed criterion of F^2^ > 2sigma(F^2^) is used only for calculating -R-factor-obs etc. and is not relevant to the choice of reflections for refinement. R-factors based on F^2^ are statistically about twice as large as those based on F, and R-factors based on ALL data will be even larger.

### Fractional atomic coordinates and isotropic or equivalent isotropic displacement parameters (Å^2^)

**Table:**

	*x*	*y*	*z*	*U*_iso_*/*U*_eq_	
Br9	0.72841 (5)	0.01588 (4)	0.86915 (2)	0.0731 (2)	
S10	0.09376 (11)	0.21269 (7)	0.99530 (4)	0.0494 (3)	
O2	0.4327 (3)	−0.12202 (18)	0.90398 (11)	0.0548 (8)	
O15	0.2816 (3)	0.53731 (17)	0.97428 (10)	0.0420 (7)	
N14	0.3916 (3)	0.35886 (16)	0.95950 (10)	0.0418 (8)	
C1	0.3047 (3)	−0.20519 (16)	0.91627 (10)	0.0621 (15)	
C3	0.4122 (4)	−0.0060 (2)	0.91890 (15)	0.0399 (10)	
C4	0.2747 (4)	0.0385 (3)	0.94491 (15)	0.0412 (10)	
C5	0.2640 (4)	0.1590 (2)	0.95831 (14)	0.0383 (10)	
C6	0.3919 (3)	0.2370 (2)	0.94450 (14)	0.0377 (9)	
C7	0.5283 (4)	0.1922 (3)	0.91780 (15)	0.0424 (10)	
C8	0.5387 (4)	0.0733 (3)	0.90492 (15)	0.0435 (10)	
C11	−0.0156 (4)	0.3122 (3)	0.93772 (15)	0.0460 (10)	
C12	0.0909 (4)	0.4072 (3)	0.90861 (14)	0.0386 (9)	
C13	0.2596 (3)	0.4382 (2)	0.95076 (14)	0.0353 (9)	
C16	−0.0182 (4)	0.5206 (3)	0.89941 (17)	0.0481 (11)	
C17	−0.1953 (5)	0.5099 (3)	0.8591 (2)	0.0743 (16)	
C18	−0.2889 (6)	0.6281 (5)	0.8496 (2)	0.0915 (19)	
C19	−0.2269 (11)	0.7005 (6)	0.8047 (4)	0.165 (4)	
C20	0.1333 (4)	0.3605 (3)	0.84640 (14)	0.0467 (11)	
C21	0.2456 (6)	0.4427 (4)	0.81384 (18)	0.0743 (16)	
C22	0.2854 (7)	0.3940 (6)	0.7533 (2)	0.105 (2)	
C23	0.3966 (11)	0.4756 (7)	0.7214 (3)	0.165 (4)	
H1A	0.30480	−0.21090	0.95970	0.0940*	
H1B	0.33020	−0.28160	0.90040	0.0940*	
H1C	0.19260	−0.17920	0.89700	0.0940*	
H4	0.18770	−0.01270	0.95360	0.0490*	
H7	0.61460	0.24340	0.90840	0.0510*	
H11A	−0.07390	0.26350	0.90470	0.0550*	
H11B	−0.10520	0.35340	0.95590	0.076 (12)*	
H14	0.48930	0.38680	0.97670	0.061 (10)*	
H16A	0.04900	0.58050	0.88150	0.0580*	
H16B	−0.03720	0.54940	0.93940	0.053 (10)*	
H17A	−0.17880	0.47760	0.81960	0.0890*	
H17B	−0.26710	0.45470	0.87810	0.0890*	
H18A	−0.41170	0.61340	0.83740	0.1100*	
H18B	−0.27580	0.67080	0.88820	0.1100*	
H19A	−0.12380	0.74130	0.82270	0.2470*	
H19B	−0.31420	0.75760	0.78950	0.2470*	
H19C	−0.20070	0.65160	0.77150	0.2470*	
H20A	0.19210	0.28460	0.85320	0.0560*	
H20B	0.02520	0.34660	0.81960	0.0560*	
H21A	0.18690	0.51850	0.80660	0.0890*	
H21B	0.35400	0.45680	0.84050	0.0890*	
H22A	0.34410	0.31820	0.76050	0.1260*	
H22B	0.17710	0.38000	0.72660	0.1260*	
H23A	0.32730	0.54030	0.70310	0.2480*	
H23B	0.44400	0.43230	0.69000	0.2480*	
H23C	0.48940	0.50610	0.75050	0.2480*	

### Atomic displacement parameters (Å^2^)

**Table:**

	*U*^11^	*U*^22^	*U*^33^	*U*^12^	*U*^13^	*U*^23^
Br9	0.0585 (3)	0.0543 (3)	0.1145 (4)	0.0039 (2)	0.0399 (2)	−0.0172 (2)
S10	0.0463 (5)	0.0452 (5)	0.0608 (6)	−0.0023 (3)	0.0220 (4)	−0.0027 (4)
O2	0.0634 (15)	0.0289 (11)	0.0748 (16)	−0.0011 (10)	0.0188 (12)	−0.0065 (11)
O15	0.0329 (11)	0.0317 (11)	0.0600 (14)	−0.0040 (8)	0.0020 (10)	−0.0114 (10)
N14	0.0289 (13)	0.0310 (13)	0.0642 (17)	−0.0052 (10)	0.0019 (12)	−0.0106 (12)
C1	0.077 (3)	0.0328 (18)	0.076 (3)	−0.0101 (17)	0.009 (2)	−0.0023 (17)
C3	0.0439 (17)	0.0286 (15)	0.0460 (19)	−0.0026 (12)	0.0022 (14)	−0.0037 (13)
C4	0.0390 (16)	0.0333 (16)	0.052 (2)	−0.0074 (12)	0.0092 (14)	−0.0011 (14)
C5	0.0352 (16)	0.0348 (16)	0.0456 (18)	−0.0019 (12)	0.0082 (13)	−0.0042 (13)
C6	0.0296 (14)	0.0308 (15)	0.0513 (19)	−0.0023 (11)	0.0010 (13)	−0.0054 (13)
C7	0.0292 (15)	0.0367 (16)	0.062 (2)	−0.0051 (12)	0.0090 (14)	−0.0041 (15)
C8	0.0360 (16)	0.0391 (17)	0.057 (2)	0.0014 (13)	0.0125 (14)	−0.0067 (15)
C11	0.0302 (15)	0.0442 (17)	0.064 (2)	−0.0063 (13)	0.0080 (15)	−0.0109 (16)
C12	0.0308 (15)	0.0375 (16)	0.0469 (18)	−0.0047 (12)	0.0033 (13)	−0.0078 (14)
C13	0.0297 (14)	0.0309 (15)	0.0464 (18)	−0.0038 (11)	0.0097 (13)	−0.0034 (13)
C16	0.0361 (17)	0.0452 (18)	0.061 (2)	−0.0002 (13)	−0.0003 (16)	−0.0098 (16)
C17	0.050 (2)	0.064 (3)	0.099 (3)	0.0067 (18)	−0.023 (2)	−0.008 (2)
C18	0.072 (3)	0.098 (4)	0.093 (3)	0.040 (3)	−0.028 (3)	−0.029 (3)
C19	0.200 (8)	0.089 (4)	0.193 (8)	0.039 (5)	−0.013 (7)	0.019 (5)
C20	0.0398 (17)	0.0490 (19)	0.050 (2)	−0.0056 (14)	0.0020 (14)	−0.0106 (15)
C21	0.083 (3)	0.088 (3)	0.055 (2)	−0.022 (2)	0.021 (2)	−0.006 (2)
C22	0.108 (4)	0.159 (5)	0.052 (3)	−0.046 (4)	0.026 (3)	−0.017 (3)
C23	0.151 (7)	0.270 (10)	0.081 (4)	−0.078 (6)	0.037 (4)	−0.028 (5)

### Geometric parameters (Å, º)

**Table:**

Br9—C8	1.885 (3)	C1—H1A	0.9600
S10—C5	1.759 (3)	C1—H1B	0.9600
S10—C11	1.811 (3)	C1—H1C	0.9600
O2—C1	1.421 (3)	C4—H4	0.9300
O2—C3	1.361 (3)	C7—H7	0.9300
O15—C13	1.231 (3)	C11—H11A	0.9700
N14—C6	1.410 (3)	C11—H11B	0.9700
N14—C13	1.354 (3)	C16—H16A	0.9700
N14—H14	0.8600	C16—H16B	0.9700
C3—C4	1.378 (4)	C17—H17A	0.9700
C3—C8	1.395 (4)	C17—H17B	0.9700
C4—C5	1.393 (4)	C18—H18A	0.9700
C5—C6	1.393 (4)	C18—H18B	0.9700
C6—C7	1.381 (4)	C19—H19A	0.9600
C7—C8	1.372 (5)	C19—H19B	0.9600
C11—C12	1.547 (5)	C19—H19C	0.9600
C12—C13	1.537 (4)	C20—H20A	0.9700
C12—C16	1.530 (5)	C20—H20B	0.9700
C12—C20	1.547 (4)	C21—H21A	0.9700
C16—C17	1.535 (5)	C21—H21B	0.9700
C17—C18	1.517 (6)	C22—H22A	0.9700
C18—C19	1.418 (9)	C22—H22B	0.9700
C20—C21	1.521 (6)	C23—H23A	0.9600
C21—C22	1.515 (6)	C23—H23B	0.9600
C22—C23	1.503 (10)	C23—H23C	0.9600
			
C5—S10—C11	101.39 (14)	S10—C11—H11B	107.00
C1—O2—C3	118.4 (2)	C12—C11—H11A	107.00
C6—N14—C13	129.4 (2)	C12—C11—H11B	107.00
C13—N14—H14	115.00	H11A—C11—H11B	107.00
C6—N14—H14	115.00	C12—C16—H16A	108.00
C4—C3—C8	118.3 (3)	C12—C16—H16B	108.00
O2—C3—C4	125.0 (3)	C17—C16—H16A	108.00
O2—C3—C8	116.7 (3)	C17—C16—H16B	108.00
C3—C4—C5	121.0 (3)	H16A—C16—H16B	107.00
S10—C5—C4	120.3 (2)	C16—C17—H17A	109.00
S10—C5—C6	119.61 (18)	C16—C17—H17B	109.00
C4—C5—C6	120.1 (3)	C18—C17—H17A	109.00
N14—C6—C5	122.4 (2)	C18—C17—H17B	109.00
C5—C6—C7	118.7 (2)	H17A—C17—H17B	108.00
N14—C6—C7	118.8 (2)	C17—C18—H18A	109.00
C6—C7—C8	121.1 (3)	C17—C18—H18B	109.00
Br9—C8—C3	119.5 (2)	C19—C18—H18A	109.00
Br9—C8—C7	119.6 (2)	C19—C18—H18B	109.00
C3—C8—C7	120.9 (3)	H18A—C18—H18B	108.00
S10—C11—C12	119.4 (2)	C18—C19—H19A	110.00
C11—C12—C20	109.1 (3)	C18—C19—H19B	109.00
C13—C12—C16	107.5 (3)	C18—C19—H19C	109.00
C13—C12—C20	109.9 (2)	H19A—C19—H19B	109.00
C16—C12—C20	110.5 (3)	H19A—C19—H19C	109.00
C11—C12—C16	108.1 (3)	H19B—C19—H19C	110.00
C11—C12—C13	111.6 (2)	C12—C20—H20A	109.00
O15—C13—C12	121.0 (2)	C12—C20—H20B	109.00
O15—C13—N14	118.7 (2)	C21—C20—H20A	109.00
N14—C13—C12	120.1 (2)	C21—C20—H20B	109.00
C12—C16—C17	116.6 (3)	H20A—C20—H20B	108.00
C16—C17—C18	112.7 (3)	C20—C21—H21A	109.00
C17—C18—C19	113.3 (5)	C20—C21—H21B	109.00
C12—C20—C21	114.9 (3)	C22—C21—H21A	109.00
C20—C21—C22	113.5 (4)	C22—C21—H21B	109.00
C21—C22—C23	113.3 (5)	H21A—C21—H21B	108.00
O2—C1—H1A	109.00	C21—C22—H22A	109.00
O2—C1—H1B	109.00	C21—C22—H22B	109.00
O2—C1—H1C	109.00	C23—C22—H22A	109.00
H1A—C1—H1B	109.00	C23—C22—H22B	109.00
H1A—C1—H1C	109.00	H22A—C22—H22B	108.00
H1B—C1—H1C	110.00	C22—C23—H23A	109.00
C3—C4—H4	119.00	C22—C23—H23B	109.00
C5—C4—H4	119.00	C22—C23—H23C	110.00
C6—C7—H7	120.00	H23A—C23—H23B	109.00
C8—C7—H7	119.00	H23A—C23—H23C	110.00
S10—C11—H11A	107.00	H23B—C23—H23C	109.00
			
C11—S10—C5—C4	−117.4 (3)	C5—C6—C7—C8	0.0 (5)
C11—S10—C5—C6	65.0 (3)	C6—C7—C8—Br9	−179.4 (2)
C5—S10—C11—C12	−51.1 (3)	C6—C7—C8—C3	−0.5 (5)
C1—O2—C3—C4	−0.6 (5)	S10—C11—C12—C13	−23.9 (4)
C1—O2—C3—C8	−178.7 (3)	S10—C11—C12—C16	−142.0 (2)
C13—N14—C6—C5	−47.0 (4)	S10—C11—C12—C20	97.8 (3)
C13—N14—C6—C7	136.2 (3)	C11—C12—C13—O15	−113.0 (3)
C6—N14—C13—O15	170.9 (3)	C11—C12—C13—N14	71.2 (3)
C6—N14—C13—C12	−13.2 (4)	C16—C12—C13—O15	5.4 (4)
O2—C3—C4—C5	−179.6 (3)	C16—C12—C13—N14	−170.4 (3)
C8—C3—C4—C5	−1.5 (5)	C20—C12—C13—O15	125.7 (3)
O2—C3—C8—Br9	−1.6 (4)	C20—C12—C13—N14	−50.0 (4)
O2—C3—C8—C7	179.5 (3)	C11—C12—C16—C17	−58.6 (4)
C4—C3—C8—Br9	−179.9 (2)	C13—C12—C16—C17	−179.2 (3)
C4—C3—C8—C7	1.2 (5)	C20—C12—C16—C17	60.8 (4)
C3—C4—C5—S10	−176.5 (3)	C11—C12—C20—C21	−177.2 (3)
C3—C4—C5—C6	1.1 (5)	C13—C12—C20—C21	−54.5 (4)
S10—C5—C6—N14	0.5 (4)	C16—C12—C20—C21	64.1 (4)
S10—C5—C6—C7	177.3 (2)	C12—C16—C17—C18	−176.6 (3)
C4—C5—C6—N14	−177.2 (3)	C16—C17—C18—C19	79.1 (6)
C4—C5—C6—C7	−0.3 (5)	C12—C20—C21—C22	179.7 (3)
N14—C6—C7—C8	177.0 (3)	C20—C21—C22—C23	−179.9 (5)

### Hydrogen-bond geometry (Å, º)

**Table:**

*D*—H···*A*	*D*—H	H···*A*	*D*···*A*	*D*—H···*A*
N14—H14···O15^i^	0.86	2.13	2.985 (3)	175
C11—H11*B*···O15^ii^	0.97	2.52	3.473 (4)	166

Symmetry codes: (i) −*x*+1, −*y*+1, −*z*+2; (ii) −*x*, −*y*+1, −*z*+2.
